# Acupuncture Attenuates Inflammation in Microglia of Vascular Dementia Rats by Inhibiting miR-93-Mediated TLR4/MyD88/NF-*κ*B Signaling Pathway

**DOI:** 10.1155/2020/8253904

**Published:** 2020-08-11

**Authors:** Lu Wang, Jing-Wen Yang, Li-Ting Lin, Jin Huang, Xue-Rui Wang, Xin-Tong Su, Yan Cao, Marc Fisher, Cun-Zhi Liu

**Affiliations:** ^1^Acupuncture Research Center, School of Acupuncture-Moxibustion and Tuina, Beijing University of Chinese Medicine, Beijing 100029, China; ^2^Department of Acupuncture and Moxibustion, Beijing Hospital of Traditional Chinese Medicine Affiliated to Capital Medical University, Beijing 100010, China; ^3^Department of Neurology, Beth Israel Deaconess Medical Center and Harvard Medical School, Boston, 02115 MA, USA

## Abstract

**Background:**

It is widely accepted that inflammation may contribute to cognitive impairment in patients with vascular dementia (VD). Our prior clinical researches have reported that acupuncture can alleviate cognitive function in VD, but the underlying mechanisms are still unclear. The purpose of this research was to explore whether acupuncture alleviates cognitive impairment by suppressing the microRNA-93- (miR-93-) mediated Toll-like receptor (TLR) signaling pathway, which triggers inflammatory responses in the central nervous system.

**Methods:**

VD was established by permanent bilateral common carotid artery occlusion in male Wistar rats. Three days after operation, the rats began daily treatment with acupuncture for two weeks. The levels of miR-93, Toll-like receptors (TLR2 and TLR4), intracellular signaling molecules (myeloid differentiation factor 88 (MyD88) and nuclear factor-kappa B (NF-*κ*B)), and inflammatory cytokines were subsequently detected. TLR4 colocalized with neurons, microglia, and astrocytes in the hippocampus was evaluated. Neuroinflammation and cognitive function were determined after intracerebroventricular injection of TLR4 antagonist TAK-242 or agonist lipopolysaccharide (LPS) with or without acupuncture.

**Results:**

We found that acupuncture notably repressed the expression of inflammatory cytokines in the hippocampus and plasma of VD rats. The expression of TLR4, but not TLR2, was markedly downregulated by acupuncture, accompanied by a decrease in miR-93 and MyD88/NF-*κ*B signaling pathway activation. The overexpression of TLR4 in microglia, but not in astrocytes and neurons, was reversed by acupuncture. Furthermore, intracerebroventricular injection of TAK-242 had similar effects to acupuncture on inflammation and cognitive function, while LPS injection abolished the beneficial effects of acupuncture.

**Conclusions:**

Taken together, these findings provide evidence that acupuncture attenuates cognitive impairment associated with inflammation through inhibition of the miR-93-mediated TLR4/MyD88/NF-*κ*B signaling pathway in experimental VD. Acupuncture serves as a promising alternative therapy and may be an underlying TLR4 inhibitor for the treatment of VD.

## 1. Introduction

Vascular dementia (VD) is the second most common type of dementia, which accounts for about 17 million cases of dementia at an annual cost of up to $200 billion [1]. Chronic cerebral hypoperfusion (CCH) is the main cause of VD, which leads to endothelial dysfunction, increases blood-brain barrier (BBB) permeability, and extravasation of plasma proteins into the brain, resulting in a strong inflammatory response [2–4]. Animal studies have confirmed that microglia are activated after the onset of cerebral hypoperfusion, leading to an early neuroinflammatory response in the brain [5, 6]. Blood samples from VD patients also showed higher levels of inflammatory molecules [7]. Therefore, inflammation is a crucial contributing factor in the development of VD.

Toll-like receptors (TLRs) are significant pattern recognition receptors of the innate immune system, which initiates inflammatory cascades by recognizing pathogen- and damaged-associated molecular patterns [8]. Toll-like receptor 2 (TLR2) and TLR4 are the two most important members of the TLR family. Prior researches have indicated that TLR2 and TLR4 take a pivotal part in inflammation after ischemic brain injury [9]. Generally, TLR2 and TLR4 can be activated by numerous extraneous and endogenous danger signaling molecules [10, 11]. Upon activation, they transmit signals through the myeloid differentiation factor 88- (MyD88-) dependent pathway to activate different transcription factors including nuclear factor-kappa B (NF-*κ*B), which eventually promotes the generation of inflammatory cytokines, such as interleukin-6 (IL-6) and tumor necrosis factor-alpha (TNF-*α*) [12]. Belkhelfa et al. [13] indicated that the TLR4 pathway induced the production of inflammatory cytokines, leading to the evolution of cerebrovascular disease during VD. Rangasamy and his colleagues [14] demonstrated that selective disruption of the TLR2-MyD88 interaction inhibits inflammation and improved memory and learning ability of the mice. Accordingly, alleviating neuroinflammation via inhibiting the TLR2 or TLR4 pathway may be an effective strategy to VD.

MicroRNAs (miRNAs) are a class of endogenous noncoding, single-stranded RNAs with 19–25 nucleotides that inhibit protein translation, degrade mRNA by combining with the 3′-untranslated regions (UTRs) of messenger RNAs (mRNAs), or activate gene expression by directly targeting the promoter sequence [15–18]. As a key immunomodulator, miRNAs take a crucial part in modulating TLR signaling pathways and innate immune responses [19]. Notably, microRNA-93 (miR-93) was implicated in the pathogenesis of VD [20, 21]. It has been reported that miR-93 is markedly upregulated in the rat cerebral cortex after cerebral ischemia [22]. In addition, one previous study revealed that miR-93 was increased in the serum of VD patients [21], implying that miR-93 may act as a potential therapeutic target for VD. Recently, lots of reports have indicated that miR-93 regulates inflammatory response by modulating TLR signaling pathways [23, 24]. Ding et al. reported that miR-93 regulated inflammation in osteoarthritis by targeting the TLR4/NF-*κ*B signaling pathway [25]. Shang et al. revealed that miR-93 improved neurological function and inhibited inflammation in rats with cerebral hemorrhage through the TLR4/NF-*κ*B signaling pathway [23]. Consequently, regulation of the miR-93-mediated TLR signal pathway is probably a potential mechanism for alleviating the inflammatory response of VD.

Acupuncture, a traditional Chinese medical therapy, has been used in many countries for the management of neurological diseases, such as rehabilitation after stroke and dementia [26, 27]. Our previous researches have clarified the beneficial role of acupuncture in improving learning and memory deficits of VD [27, 28]. In particular, a recent report indicated that acupuncture improved the cognitive performance of VD rats via the downregulation of the inflammatory response [29]. However, it is still not clear how acupuncture inhibits neuroinflammation in VD rats. This study was designed to verify the hypothesis that acupuncture improves cognitive impairment via modulating the miR-93-mediated TLR2/TLR4 signaling pathway to suppress the inflammatory responses.

## 2. Materials and Methods

### 2.1. Animals

Male Wistar rats (270–320 g), provided by the Vital River Laboratory (Beijing, China), were maintained with temperature (25 ± 2°C), humidity (60 ± 5%), and light (12-h light/dark cycle) control with free access to water and food. All animal experiments were conducted after being approved by the Institutional Animal Care and Use Committee of the Beijing University of Chinese Medicine.

### 2.2. Permanent Common Carotid Artery Occlusion

As we previously described, the rat model of VD was established by permanent bilateral common carotid artery occlusion (2VO) [30, 31]. Rats were anesthetized intraperitoneally with 40 mg/kg pentobarbital sodium. After the bilateral common carotid arteries were exposed through an abdominal median incision, we lightly separated them from the vagus nerve and then ligated the bilateral blood vessels with 5-0 silk thread. In the sham operation group, the same operation was performed with the exception of arterial ligation. Throughout the surgery process, the operation was as gentle as possible to reduce the pain of the animal, and a heating blanket was used to maintain the rats' body temperature.

### 2.3. Acupuncture Treatment

**Figure 1:**
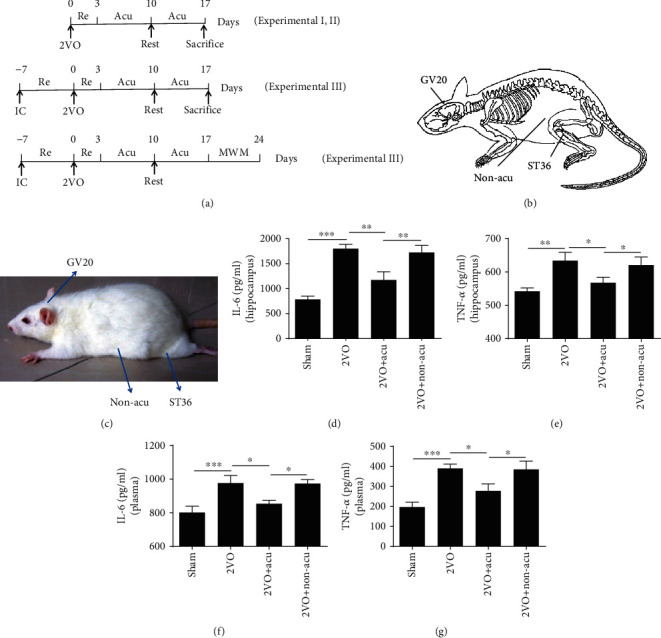
Acupuncture at Zusanli and Baihui reduces the inflammatory response in 2VO rats. (a) Protocols for acupuncture manipulation of the 2VO rats. IC: implanting cannula into the lateral ventricle; Re: recovery; Acu: acupuncture; MWM: Morris water maze. (b, c) The location of applied points in rat. (d–g) The levels of IL-6 and TNF-*α* in the hippocampus and plasma were detected at day 14 after acupuncture treatment in sham, 2VO, 2VO+Acu, and 2VO+Non-acu groups by ELISA. Values are mean ± SEM of 6 animals per group. ^∗^*P* < 0.05, ^∗∗^*P* < 0.01, and ^∗∗∗^*P* < 0.001, compared as indicated.

Three days after surgery, the animals were treated with acupuncture without anesthesia, once a day for two weeks and one day off after six treatments. After cleaning the skin with 75% alcohol, sterilized disposable stainless steel needles (0.2 × 5 mm, Huanqiu, China) were inserted 4 mm deep at the selected points. In the 2VO+Acu group, Baihui (GV20) and bilaterally Zusanli (ST36) acupoints were chosen as the acupuncture sites. In the 2VO+Non-acu group, the bilateral hypochondria, 10 mm above the anterior superior, was chosen as the insertion site. Detailed acupoint locations and manipulations are shown in Figure 1(b) and Supplemental Table 1. Ten minutes after insertion, the needles were removed. The sham group and the 2VO group were given the same time and same level catching-grasping stimulus without acupuncture treatment.

### 2.4. Experimental Design

#### 2.4.1. Experimental I

To determine the anti-inflammation effect of acupuncture, rats were randomly assigned to 4 groups: sham, 2VO, 2VO+Acu, and 2VO+Non-acu (6 rats per group). The release of inflammatory cytokines in the hippocampus and plasma was detected by enzyme-linked immunosorbent assay (ELISA) (Figure 1(a)).

#### 2.4.2. Experimental II

To observe the effect of acupuncture treatment on regulating the expressions of miR-93 and TLR signaling pathways, 48 rats were randomly assigned to 4 groups: sham, 2VO, 2VO+Acu, and 2VO+Non-acu. The expressions of miR-93, TLR2 mRNA, and TLR4 mRNA were measured by real-time PCR. The protein expressions of TLR2, TLR4, MyD88, and p-NF-*κ*B p65 were measured by Western blot. The activation of TLR4 in the hippocampus was detected by immunohistochemistry. The distribution of TLR4 colocalized with neurons, astrocytes, and microglia of the hippocampus was determined by immunofluorescence (Figure 1(a)).

#### 2.4.3. Experimental III

To assess the role of TLR4 in the mechanism of beneficial effects of acupuncture, an intracerebroventricular injection of TLR4 antagonist TAK-242 and agonist lipopolysaccharide (LPS) were performed. Rats were randomly assigned to different test agent groups with or without acupuncture and vehicle control groups: sham, 2VO, 2VO+Acu, 2VO+TAK-242, and 2VO+DMSO; sham, 2VO, 2VO+Acu, 2VO+Acu+LPS, 2VO+Acu+Saline, and 2VO+LPS groups (12 rats per group). The cognitive function and proinflammatory cytokine levels were evaluated by the Morris water maze (MWM) test and ELISA, respectively (Figure 1(a)).

### 2.5. Drug Administration

The TLR4 agonist LPS (055 : B5, Sigma-Aldrich) was dissolved in sterile saline and was administered at 1 *μ*g/10 *μ*l [32] into the lateral ventricle. The TLR4 antagonist TAK-242 (CLI-095, InvivoGen) was dissolved in 10% dimethyl sulfoxide (DMSO, D2650, Sigma-Aldrich) and was infused via intracerebroventricular injection at a dose of 0.6 mg/10 *μ*l [33, 34]. Drugs were infused by a Hamilton syringe (80630, Hamilton Co., Reno, USA) with a rate of 1 *μ*l/min, and the needle was left for an additional 10 min before withdrawal to prevent reflux. All the infusion procedures were conducted 30 min before treatment every day.

### 2.6. Intracerebroventricular

Infusion rats were anesthetized with pelltobarbitalum natricum and placed in a stereotaxic apparatus (SR-6R; Narishige, Japan). A stainless-steel cannula was implanted unilaterally into the lateral ventricle (from the bregma: anterior-posterior -0.8 mm, lateral 1.3 mm, and ventral -3.5 mm). Finally, the cannula was fixed on the skull with dental cement. Following surgery, animals were housed individually and allowed to recover for 7 days.

### 2.7. MWM

To assess spatial learning and memory, the MWM was used as described before [35]. The circular water maze pool was 160 cm in diameter and divided into 4 quadrants. In the acquisition training, animals were subjected to 4 training trials per day for 5 consecutive days. Animals were allowed 90 s to find the platform. Upon failure to locate the submerged platform, animals were guided to the platform and allowed to stay there for 10 s. On day 6, a probe trail was conducted by removing the platform from the pool. The animals were left to swim freely for 90 s. Time latency, swimming speed, and percentage time spent in the target quadrant were recorded. During experiments and analysis, the investigators were blinded to the experimental group. Animals were identified by earmarks and had numbers, which were announced to the investigator only after finishing experiments and analysis.

### 2.8. ELISA

Supernatants from the hippocampus and plasma homogenates were used for detecting the concentrations of IL-6 and TNF-*α* with commercially available ELISA kits (Boshide, Wuhan, China) according to the manufacturer instructions.

### 2.9. Western Blot

The protein concentrations in the hippocampus were determined by the Pierce BCA Protein Assay kit (Thermo Scientific). Samples were separated by 8-12% sodium dodecyl sulfate-polyacrylamide gel electrophoresis (SDS-PAGE) (Applygen) and electrically transferred onto polyvinylidene fluoride (PVDF) membranes. The membranes were blocked with 5% nonfat dried milk for 1 h and incubated overnight at 4°C with primary antibodies directed against TLR2, TLR4, MyD88, NF-*κ*B p65, p-NF-*κ*B p65, and *β*-actin (Supplement Table 2). The membranes were incubated with secondary antibodies for 1 h at room temperature and visualized using the Odyssey Infrared Imaging System (LI-COR Biosciences, Nebraska, USA).

### 2.10. Immunohistochemistry and Immunofluorescence

For immunohistochemistry staining, the brain tissues from rats were collected after completion of the acupuncture treatment protocol and were frozen and sectioned with a cryostat microtome (20 *μ*m). Brain sections with antigen retrieved in citric acid were incubated with a primary antibody to TLR4 (Supplemental Table 3) overnight at 4°C after blocking with goat serum, then incubated with secondary antibodies for 1 h and detected with 3,3′-diaminobenzidine (DAB). Images were analyzed by a researcher who was blind to the assignment group using Image-Pro Plus software. For immunofluorescence staining, brain sections were blocked with goat serum and then incubated with primary antibodies overnight at 4°C to detect the colocalization between TLR4 and neurons (NeuN), TLR4 and microglia (Iba1), and TLR4 and astroglia (GFAP) (Supplemental Table 3). Subsequently, sections were washed and incubated with second antibodies for 1 h and examined after mounting with 4′,6′,-diamidino-2-phenylindole (DAPI).

### 2.11. RNA Extraction and Real-Time PCR

Total RNA containing miRNAs and mRNAs was extracted from the hippocampus using a TRIzol reagent (Introgen, Carlsbad, CA) following the manufacturer instructions. Complementary DNA (cDNA) was synthesized with miRNA First Strand cDNA Synthesis Kit (Tailing Reaction) (Sangon Biotech) and RNA PCR kit (Promega, Madison, WI). The miRNA and mRNA levels of genes of interest were analyzed by real-time PCR, which was performed with 2x SYBR master mix (Takara, Otsu, Shiga, Japan), using a Bio-Rad iCycler iQ5 (Bio-Rad, Hercules, CA). The PCR cycle was as follows: 95°C/30 s, 40 cycles of 94°C/30 s, 59°C/30 s, and 72°C/1 min, and the melt-curve analysis was performed following each experiment. U6 RNA included in the kit was used as an endogenous reference gene for normalizing the miR-93 gene expression. Gene mRNA levels were normalized to that of GAPDH. The results were calculated by the 2^−*ΔΔ*Ct^ method. The sequences of the U6 RNA and the universal PCR reverse primer are proprietary information held by Sangon Biotech. Primers for miR-93, TLR2, TLR4, and GAPDH are listed in Supplemental Table 4.

### 2.12. Statistical Analysis

Data were expressed as mean ± SEM. MWM data were analyzed using two-way repeated measure ANOVA. One-way ANOVA was conducted to compare the results of quantitative data of the Western blot, ELISA, PCR analysis, and immunohistochemistry with the SPSS 22.0 software (IBM, Armonk, NY). *P* values < 0.05 were considered statistically significant.

## 3. Results

### 3.1. Acupuncture Reduces Inflammatory Response in 2VO Rats

In order to identify the effect of acupuncture on the 2VO-induced inflammatory response in the hippocampus and plasma, we detected inflammatory mediators including IL-6 and TNF-*α*. The results showed that IL-6 were increased after 2VO surgery (*P* < 0.001), while the increase was suppressed by acupuncture both in the hippocampus and plasma (*P* < 0.01 and *P* < 0.05, resp.) (Figures 1(d) and 1(f)). Similarly, the TNF-*α* level of the hippocampus and plasma in the 2VO group was evidently higher than that in the sham group (*P* < 0.01 and *P* < 0.001, resp.), while acupuncture treatment could significantly reverse the overexpression of TNF-*α* induced by 2VO operation (*P* < 0.05) (Figures 1(e) and 1(g)). However, significant difference was not observed between the 2VO and 2VO+Non-acu groups (*P* > 0.05) (Figures 1(d)–1(g)).

### 3.2. Acupuncture Inhibits the Expression of TLR4, but Not TLR2, in the Hippocampus of 2VO Rats

**Figure 2:**
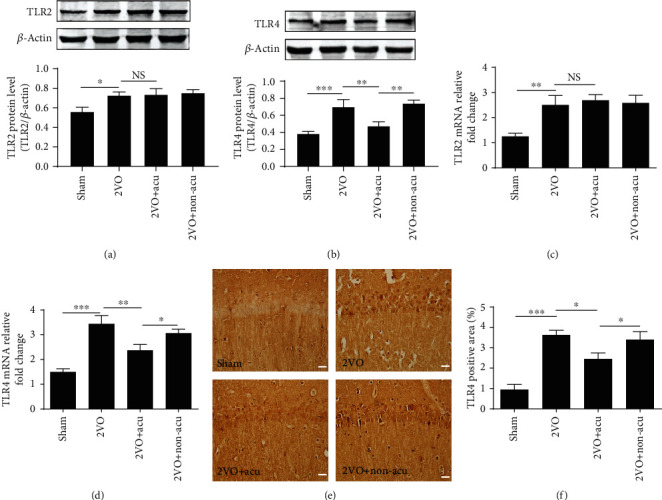
Acupuncture inhibits the expression of TLR4, but not TLR2, in the hippocampus of 2VO rats. (a, b) Representative Western blots and the densitometric analysis of TLR2, TLR4, and its corresponding *β*-actin bands in protein levels were determined at day 14 after acupuncture treatment using Western blot analysis. The lanes (from left to right) represent the sham, 2VO, 2VO+Acu, and 2VO+Non-acu groups (*n* = 6). (c, d) The mRNA expressions of TLR2 and TLR4 in the hippocampus were assessed by quantitative real-time PCR at day 14 after acupuncture treatment in the sham, 2VO, 2VO+Acu, 2VO+Non-acu groups (*n* = 6). (e) Representative photomicrographs of TLR4 immunohistochemistry are shown. (f) Graphic presentations show the positive area of TLR4 in the hippocampal CA1 region at day 14 after acupuncture treatment (*n* = 6). ^∗^*P* < 0.05, ^∗∗^*P* < 0.01, and ^∗∗∗^*P* < 0.001, compared as indicated.

We evaluated whether acupuncture could exert a central anti-inflammatory effect associated with downregulating TLRs in the hippocampus. The protein and mRNA levels of TLR4 were increased in the 2VO group compared with the sham group (*P* < 0.001); in contrast, rats in the 2VO+Acu group showed obviously reduced protein and mRNA levels of TLR4 in comparison to those in the 2VO group (*P* < 0.01); no significant difference was found between the 2VO and 2VO+Non-acu groups (*P* > 0.05) (Figures 2(b) and 2(d)). Unexpectedly, acupuncture did not affect the protein and mRNA expression of TLR2 in the hippocampus of 2VO rats (*P* > 0.05) (Figures 2(a) and 2(c)). Immunohistochemical staining of the hippocampus showed similar results as the Western blot. The activation of TLR4 was significantly increased in 2VO rats (*P* < 0.001), while decreased in the 2VO+Acu group (*P* < 0.05); there was no significant difference between the 2VO and 2VO+Non-acu groups (*P* > 0.05) (Figures 2(e) and 2(f)).

### 3.3. Acupuncture Reduces the Expression of TLR4 in Microglia

**Figure 3:**
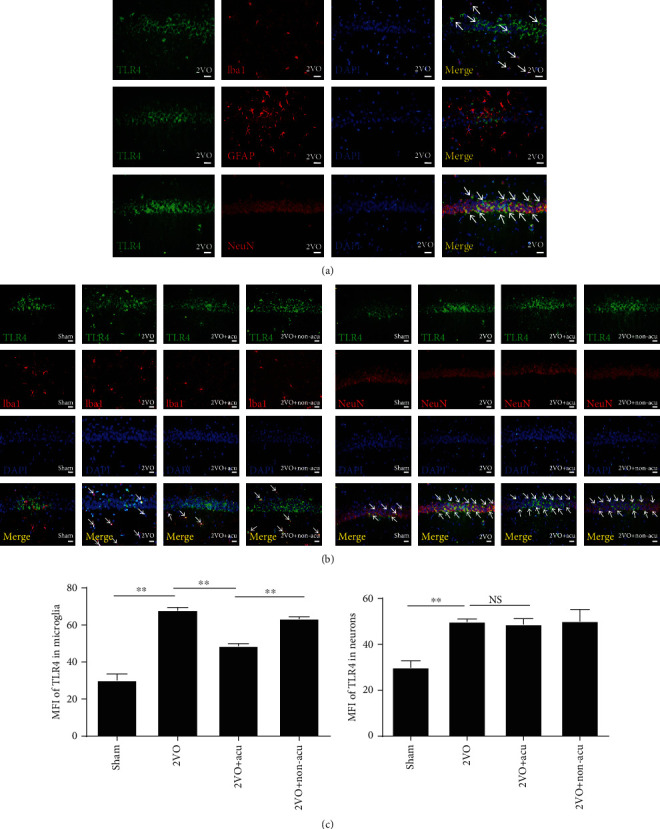
Acupuncture reduces the expression of TLR4 in microglia. (a) Representative photomicrographs of TLR4 (green) colocalized with neurons (NeuN, red), microglia (Iba1, red), and astroglia (GFAP, red) in the hippocampus of 2VO rats are shown. Nuclei were stained with DAPI (blue). (b) Representative photomicrographs of TLR4 immunofluorescence are shown for each condition. TLR4 localizes to the respective cellular marker with areas of overlap appearing yellow in the merged image (white arrow). Scale bars: 50 *μ*m. (c) The graph shows the MFI of TLR4 in microglia or neurons in the hippocampus of 2VO rats (*n* = 6). ^∗∗^*P* < 0.01, compared as indicated. MFI: mean fluorescence intensity; NS: no significance.

We further examined which cell types in the hippocampus were associated with the expression of TLR4. The results revealed that TLR4 was mainly colocalized within microglia and neurons, but not within astroglia (Figure 3(a)). TLR4 immunoreactivity in microglia significantly increased after 2VO surgery (*P* < 0.01), while acupuncture ameliorated the enhancement (*P* < 0.01) (Figures 3(b) and 3(c)). No major differences in TLR4 expression were found between the 2VO and 2VO+Non-acu groups (*P* > 0.05) (Figures 3(b) and 3(c)). In addition, 2VO surgery induced enhanced TLR4 immunoreactivity in neurons (*P* < 0.01), but there was no significant difference between the 2VO and 2VO+Acu groups (*P* > 0.05) (Figures 3(b) and 3(c)).

### 3.4. Acupuncture Suppresses the Activation of MyD88/NF-*κ*B Signaling Pathway

**Figure 4:**
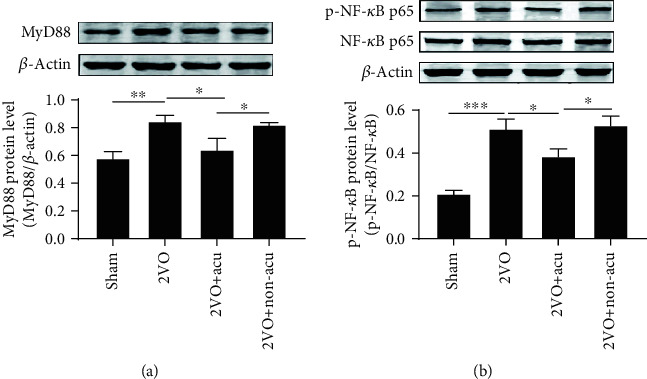
Acupuncture suppresses the expressions of MyD88 and NF-*κ*B in the hippocampus of 2VO rats. (a, b) The protein levels of MyD88, NF-*κ*B, p-NF-*κ*B, and its corresponding *β*-actin bands in the hippocampus were determined in sham, 2VO, 2VO+Acu, and 2VO+Non-acu groups using Western blot (*n* = 6). ^∗^*P* < 0.05, ^∗∗^*P* < 0.01, and ^∗∗∗^*P* < 0.001, compared as indicated.

To explore whether the downstream pathway of TLR4 was also regulated by acupuncture, the protein expression of MyD88 and the phosphorylation level of NF-*κ*B p65 in the hippocampus were investigated. The results revealed that the protein levels of MyD88 and p-NF-*κ*B p65 were significantly elevated in the 2VO group (*P* < 0.01 and *P* < 0.001, resp.), but the increase was inhibited by acupuncture (*P* < 0.05) (Figures 4(a) and 4(b)). However, no major difference was found between the 2VO and 2VO+Non-acu groups (*P* > 0.05) (Figures 4(a) and 4(b)).

### 3.5. Acupuncture Inhibits the Level of miR-93 in the Hippocampus of 2VO Rats

**Figure 5:**
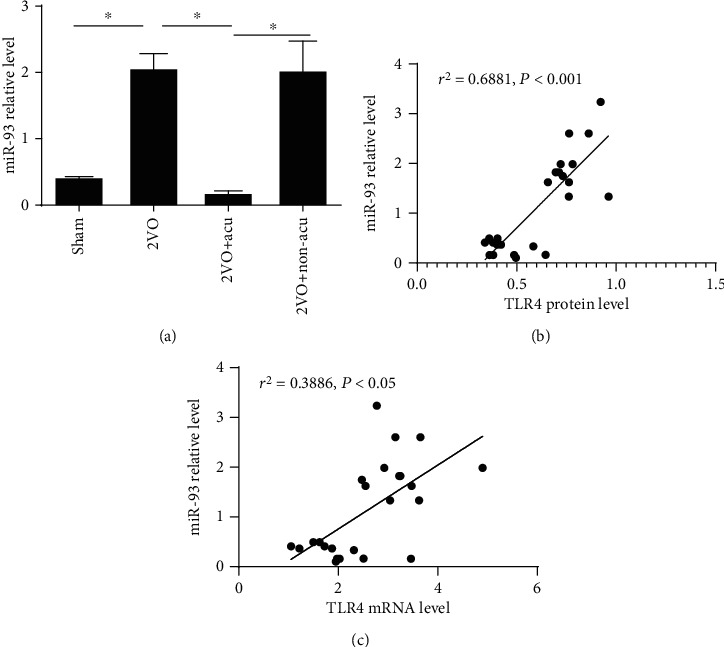
Acupuncture suppresses the level of miR-93 in the hippocampus of 2VO rats. (a) The level of miR-93 in the hippocampus was assessed by quantitative real-time PCR at day 14 after acupuncture treatment (*n* = 6). ^∗^*P* < 0.05, compared as indicated. (b) The scatter plot describing the correlation between the miR-93 relative level and the TLR4 protein level in the hippocampus, *n* = 24 for each correlation. (c) The scatter plot describing the correlation between the miR-93 relative level and the TLR4 mRNA level in the hippocampus, *n* = 24 for each correlation.

Since miR-93 may act as a potential biomarker for ischemic brain damage and is a critical player in the regulation of the TLR4 signaling pathway, we hypothesized that the inhibition of TLR4 signaling after acupuncture treatment in 2VO rats might be due to the regulation of miR-93 expression. The results showed that the level of miR-93 in the 2VO group was significantly upregulated in comparison with that in the sham group (*P* < 0.05), but acupuncture treatment notably inhibited the overexpression of miR-93 (*P* < 0.05). Conversely, there was no significant difference between the 2VO group and the 2VO+Non-acu group (*P* > 0.05) (Figure 5(a)). To assess whether miR-93 levels could influence the expression of TLR4 in the hippocampus of 2VO rats, we analyzed the relationship between miR-93 and TLR4 protein/mRNA levels in the hippocampus. Pearson's correlation analysis showed that miR-93 and TLR4 protein/mRNA levels in the hippocampus had a significant positive linear correlation (*P* < 0.001 and *P* < 0.05, resp.) (Figures 5(b) and 5(c)), implying that miR-93 may positively regulate the expression of TLR4.

### 3.6. TLR4 Antagonist Mimics the Neuroprotective Effects of Acupuncture, and TLR4 Agonist Reverses the Beneficial Effects of Acupuncture

**Figure 6:**
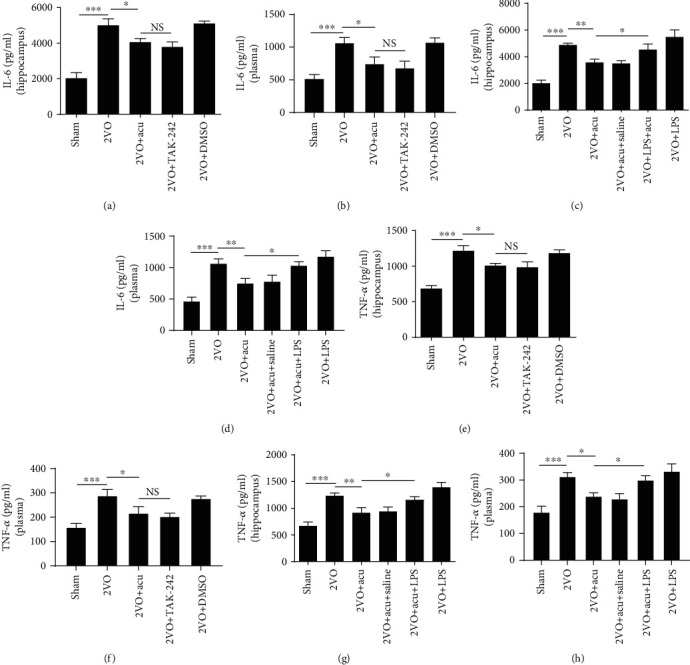
TLR4 antagonist mimics the effects of acupuncture on inflammation inhibition, and TLR4 agonist reverses these effects of acupuncture. The levels of IL-6 (a, b) and TNF-*α* (e, f) in the hippocampus and plasma were detected in sham, 2VO, 2VO+Acu, 2VO+TAK-242, and 2VO+DMSO groups by ELISA (*n* = 6). The levels of IL-6 (c, d) and TNF-*α* (g, h) in the hippocampus and plasma were detected in sham, 2VO, 2VO+Acu, 2VO+Acu+Saline, 2VO+Acu+LPS, 2VO+LPS groups by ELISA (*n* = 6). ^∗^*P* < 0.05, ^∗∗^*P* < 0.01, and ^∗∗∗^*P* < 0.001, compared as indicated. NS: no significance.

**Figure 7:**
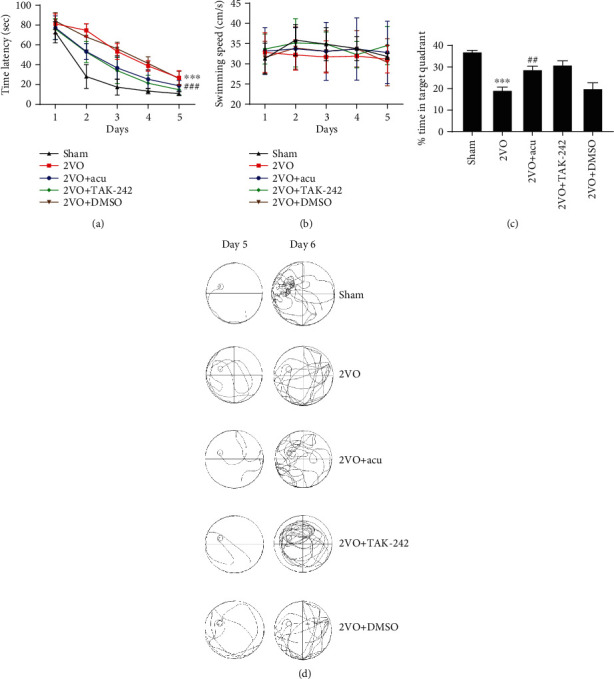
TLR4 antagonist mimics the effects of acupuncture on cognitive improvement. (a) Time latency from the first day to the fifth, (b) average swimming speed of rats on acquisition training, and (c) percentage of time spent in the target quadrant on the probe trial were analyzed in sham, 2VO, 2VO+Acu, 2VO+TAK-242, and 2VO+DMSO groups using the Morris water maze. (d) Typical swimming traces were recorded on day 5 and day 6 among these groups. Values are mean ± SEM of 6 animals per group. ^∗∗∗^*P* < 0.001 2VO vs. sham group; ^##^*P* < 0.01 and ^###^*P* < 0.001 2VO+Acu vs. 2VO group.

**Figure 8:**
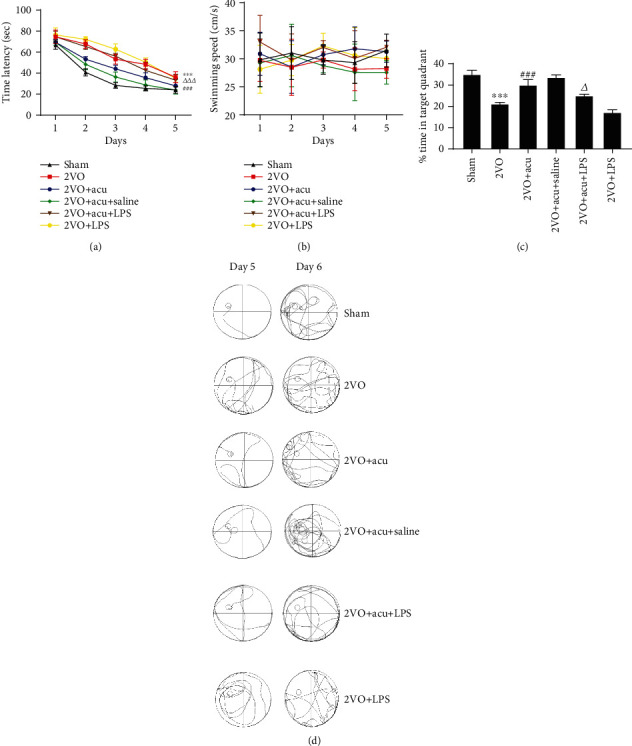
TLR4 agonist reversed these effects of acupuncture on cognitive improvement. (a) Time latency from the first day to the fifth, (b) average swimming speed of rats on acquisition training, and (c) percentage of time spent in the target quadrant on the probe trial were analyzed in sham, 2VO, 2VO+Acu, 2VO+Acu+Saline, 2VO+Acu+LPS, and 2VO+LPS groups using the Morris water maze. (d) Typical swimming traces were recorded on day 5 and day 6 among these groups. Values are mean ± SEM of 6 animals per group. ^∗∗∗^*P* < 0.001 2VO vs. sham group; ^###^*P* < 0.001 2VO+Acu vs. 2VO group; ^△^*P* < 0.05 and ^△△△^*P* < 0.01 2VO+Acu+LPS vs. 2VO+Acu group.

Given that the neuroprotective effects of acupuncture may be partly mediated by TLR4, we speculated that a TLR4 antagonist may have similar effects as acupuncture on attenuating inflammation and improving cognitive competence. The TLR4 agonist may mitigate the beneficial effects of acupuncture. The levels of IL-6 and TNF-*α* in the hippocampus and plasma were significantly alleviated in 2VO+Acu group compared with the 2VO group (*P* < 0.05 or *P* < 0.01) (Figures 6(a) – 6(h)). Nevertheless, administration LPS significantly abolished the beneficial effects of acupuncture (*P* < 0.05) (Figures 6(c)–6(d) and 6(g)–6(h)). Significant difference was not found between the 2VO+TAK-242 and 2VO+Acu groups, the 2VO and 2VO+DMSO groups, and the 2VO+Acu and 2VO+Acu+Saline groups (*P* > 0.05) (Figures 6(a)–6(h)). To evaluate whether cognitive improvement in acupuncture-treated rats is related to the TLR4, rats were subjected to the MWM test after microinjection. Acupuncture treatment markedly reduced the time latency and increased % time in the target quadrant (*P* < 0.001 or *P* < 0.01) (Figures 7(a), 7(c), 8(a) and 8(c)). It is noteworthy that there were no apparent differences between the 2VO+TAK-242 and 2VO+Acu groups and the 2VO and 2VO+DMSO groups (*P* > 0.05) (Figures 7(a) and 7(c)). However, the therapeutic effect of acupuncture on time latency and % time in the target quadrant of 2VO rats was apparently abolished by LPS administration (*P* < 0.001 and *P* < 0.05, resp.) (Figures 8(a) and 8(c)). Application of saline in acupuncture-treated animals did not produce obvious alterations in spatial learning and memory compared with the 2VO+Acu group (*P* > 0.05) (Figures 8(c) and 8(c)). Additionally, there was no difference in the swimming speed among these groups (*P* > 0.05) (Figures 7(b) and 8(b)).

## 4. Discussion

In the present study, we show that acupuncture reduced the levels of inflammatory cytokines in the hippocampus and plasma of VD rats. These beneficial effects were associated with the downregulation of TLR4, but not TLR2, in the hippocampus. The overexpressed TLR4 protein is colocalized with neurons and microglia in the hippocampus. Acupuncture downregulated TLR4 expression in microglia of the hippocampus, but not in neurons. Meanwhile, the overexpression and activation of the miR-93 and MyD88/NF-*κ*B signaling pathway were reversed by acupuncture. TLR4 antagonist TAK-242 had similar effects as acupuncture on inflammation and cognitive function, while TLR4 agonist LPS inhibited the beneficial effects of acupuncture.

VD is characterized by a progressive worsening of memory and other cognitive functions resulting from cerebrovascular disorders [2]. The mechanisms that induce cognitive impairment in VD are multifaceted, such as neuronal apoptosis [36], oxidative stress [37], and neurotransmitter abnormities [38]. Recently, many studies have mentioned that inflammation is important in the development of cognitive impairment with VD [32, 39]. CCH triggers inflammatory cascades in hypoxia-ischemia brain tissue, which results in persistent functional modification of hippocampal excitatory synapses and leads to cognitive impairment [40, 41]. Acupuncture has been reported to be a possible nonpharmacological treatment for VD patients. Our previous clinical trials suggested that acupuncture is useful in relieving symptoms of VD [42]. In this study, we found that acupuncture improved cognitive performance and reduced the levels of IL-6 and TNF-*α* in the hippocampus and plasma. These results indicate that acupuncture exerts its protective effect on cognitive improvement through the inhibition of inflammation in the hippocampus.

TLRs are the primary regulators of the innate immune system and play an indispensable role in the inflammatory response caused by ischemic brain injury [9, 43]. Our results revealed that the protein and mRNA levels of TLR4 in the hippocampus were increased in 2VO rats, but the increase was reversed by acupuncture. Surprisingly, we did not find downregulation of TLR2 by acupuncture in 2VO rats, which differs from some previous studies [44, 45]. This may be due in part to the difference in the model or intervention we used. Studies have shown that TLR4 is widely expressed in neurons, microglia, astrocytes, and endothelial cells of the central nervous system (CNS) [46]. Microglia, as the first line of immune defense in the CNS, takes an important part in the neuroinflammation. TLR4 is abundantly expressed in microglia and induces the activation of microglia [47]. Activated microglia gives rise to inflammatory cascade reaction, which results in brain injury and neurological dysfunction [48, 49]. Qin et al. demonstrated that TLR4 deficiency could suppress the proinflammatory state of microglia, alleviating cognitive dysfunction in CCH mice [50]. In our experiment, we showed that TLR4 was predominantly colocated with microglia and neurons. But the expression of TLR4 in microglia, not neurons, was dramatically inhibited by acupuncture. This observation suggests that TLR4 in microglia may act as a potential target for acupuncture to modulate inflammation in VD.

TLR4 generally plays a key role in inflammatory response through the MyD88-dependent pathway [51]. MyD88 is not only a crucial downstream signaling ligand of TLR4 receptor complex but also an essential adapter protein of the NF-*κ*B signaling pathway. The NF-*κ*B pathway is the central mediator involved in immune and inflammatory responses. Once being activated, the NF-*κ*B p65 subunit translocates into the nucleus, triggering the production of inflammatory factors [52, 53]. Lan et al. reported that acupuncture treatment significantly alleviated neurological deficit scores and neuroinflammation in rats after focal cerebral ischemia-reperfusion injury, which was mediated by inhibiting the expression of ischemic cerebral tissues TLR4 and NF-*κ*B p65 [54]. Han et al. observed that acupuncture could modulate the inflammatory reaction in middle cerebral artery occlusion (MCAO) rats via suppressing the TLR4/NF-*κ*B signaling pathway in microglia [29]. In agreement with these researches, we found that the expressions of MyD88 and p-NF-*κ*B p65 were markedly enhanced in 2VO rats, while acupuncture could evidently reduce their expressions. Our data imply that regulating the TLR4/MyD88/NF-*κ*B pathway in microglia may take part in the effect of acupuncture against neuroinflammation.

By regulating the expression of multiple genes and pathways, miRNAs play a pivotal role in the pathogenesis of many neuroinflammatory disorders [55]. Furthermore, the aberrant expression of particular miRNAs may be related with a variety of CNS diseases [56, 57]. It has been reported that the level of miR-93 was significantly higher in the VD patients [21] and MCAO-induced ischemic stroke mice [22], implying that miR-93 may be a potential biomarker for ischemia brain damage. In this study, we found that the expression of miR-93 was upregulated in 2VO rats, while was reversed by acupuncture treatment. Moreover, in recent years, miR-93 has received considerable attention as a newly identified family of regulators implicated in fine-tuning the TLR4 signaling pathway. Tang et al. manifested that miR-93 regulated LPS-induced inflammation of cardiac myocytes by targeting TLR4 [58]. Liu et al. showed that miR-93 attenuated inflammation caused by acute myocardial infarction via regulating the expression of TLR4 [24]. Another research revealed that miR-93 antagomir could notably alleviate inflammation via reducing the expression of TLR4 and NF-*κ*B p65 in rats' brain tissues, indicating that miR-93 can positively regulate the TLR4 signaling pathway [23]. In line with these findings, we observed that a significant positive correlation existed between miR-93 and TLR4 levels, which implicates that miR-93 is likely to positively modulate the expression of TLR4 in hippocampus of 2VO rats. Taken together, these results suggest that the neuroinflammation of 2VO rats is probably modulated by acupuncture through inhibition of the miR-93-mediated TLR4 signaling pathway.

TAK-242 is a specific antagonist of TLR4 [59], which is able to block TLR4 signaling, mediates the expression of inflammatory cytokines, and protects the brain from damage due to cerebral ischemia [33, 60]. LPS is the most typical pathogen-associated molecular pattern of TLR4 and a strong inducer of inflammation [11, 12]. Trace amounts of LPS activate the innate immune system via TLR4, leading to the production of numerous proinflammatory mediators. Tang et al. [61] elucidated that TAK-242 treatment significantly decreased the expression of TLR4 and its downstream signal molecules in ipsilateral hippocampal lesions of rats with hypoxic-ischemic brain damage and alleviated the activation of microglia and the loss of learning and memory function. Dong et al. [62] exhibited that LPS injection obviously induced upregulation of the TLR4 signaling pathway, activation of microglia, and release of inflammatory cytokines in mice, leading to memory impairment. Consistent with these findings, we found that acupuncture mimicked the effects of TAK-242 in decreasing the inflammatory response and ameliorating the cognitive deficits. Besides, LPS administration weakened the beneficial effects of acupuncture in 2VO rats. These results demonstrate that TLR4 plays an important role in the inflammatory response of VD. The anti-inflammatory effect of acupuncture in VD is via the regulation of a key conductor, TLR4.

There are several limitations in our study. First of all, considering that LPS and TAK-242 are recognized agonist and antagonist of TLR4, we did not further detect the expression of TLR4 after use. Secondly, this study provides evidence of association not causality since we did not directly verify the relationship between miR-93 and TLR4 in the hippocampus of 2VO rats. In the future, more studies can be carried out to explore the specific mechanism of TLR4 expression induced by miR-93 in VD.

## 5. Conclusion

**Figure 9:**
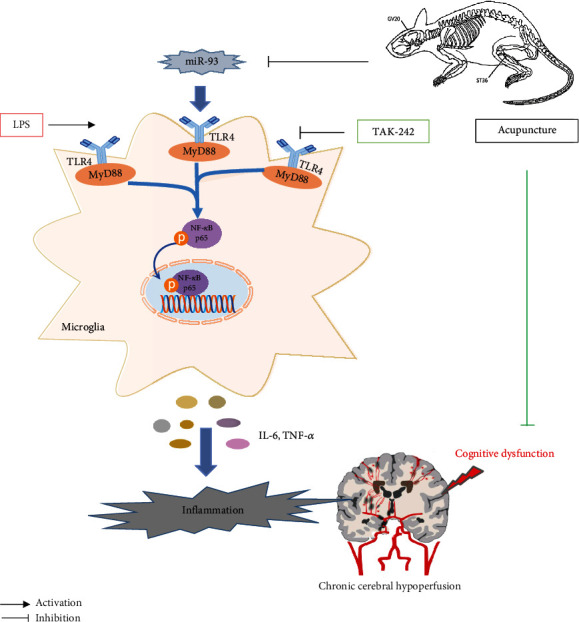
Proposed scheme showing how acupuncture inhibits 2VO-induced inflammatory response via the miR-93-mediated TLR4/MyD88/NF-*κ*B signaling pathway. Acupuncture treatment alleviates cognitive dysfunction and decreases IL-6 and TNF-*α* expressions. These effects involve suppression of miR-93 expression and the inhibition of activation of the TLR4/MyD88/NF-*κ*B signaling pathway. TLR4 antagonist TAK-242 mimics the effects of acupuncture, while TLR4 agonist LPS reverses the beneficial effects of acupuncture.

In summary, we demonstrated that acupuncture treatment alleviated inflammation in VD, which was probably related to the inhibition of the miR-93-mediated TLR4/MyD88/NF-*κ*B signaling pathway (Figure 9). In particular, acupuncture may be a potential TLR4 inhibitor for treating VD. The present investigation provides a new perspective on the anti-inflammatory mechanism of acupuncture and implicates acupuncture as a potential complementary therapy for cognitive dysfunction.

## Data Availability

The data used to support the findings of this study are available from the corresponding author upon request.

## Supplementary Materials

Supplementary materials Supplementary Table 1: acupuncture points and manipulations. Supplementary Table 2: antibodies used for Western blot. Supplementary Table 3: antibodies used for immunofluorescence. Supplementary Table 4: primer sequence for real-time PCR.
