# Supplementary material for: Acupuncture Attenuates Inflammation in Microglia of Vascular Dementia Rats by Inhibiting miR-93-Mediated TLR4/MyD88/NF-κB Signaling Pathway
Source: Oxid Med Cell Longev. 2020 Aug 11;2020:8253904. doi: 10.1155/2020/8253904 (PMC7441436; doi:10.1155/2020/8253904)
Supplement: Supplementary materials — Supplementary Table 1: acupuncture points and manipulations. Supplementary Table 2: antibodies used for Western blot. Supplementary Table 3: antibodies used for immunofluorescence. Supplementary Table 4: primer sequence for real-time PCR. [file 8253904.f1.docx]

**SUPPLEMENTARY MATERIALS**

**Supplemental Table 1**: Acupuncture points and manipulations.

| **Points** | **Anatomical positions** | **Manipulation** | **Frequency**  **(per minutes)** | **Manipulation Time**  **(min)** | **Retaining**  **Time**  **(min)** |
| --- | --- | --- | --- | --- | --- |
| **Baihui (GV20)** | The intersection of the sagittal midline and the line linking two rat ears | Twirling  reinforcing  manipulation | 120 | 1 | 9 |
| **Zusanli (ST36)** | 2 mm lateral to the anterior tubercle of the tibia in the anterior tibial muscle and 5 mm distal to the knee joint lower point | Twirling reinforcing  manipulation | 120 | 1 | 9 |
| **Non-acupoint** | Hypochondrium 10 mm above iliac crest | Without manipulation | 0 | 0 | 10 |

**Supplemental Table 2**: Antibodies used for Western blot.

| **Antibodies** | **Catalog No.** | **Dilution** | **Company** |
| --- | --- | --- | --- |
| **TLR2** | ab16894 | 1:200 | Abcam |
| **TLR4** | ab22048 | 1:200 | Abcam |
| **MyD88** | ab2064 | 1:200 | Abcam |
| **p-NF-**κ**B p65** | #3039 | 1:250 | CST |
| **NF-**κ**B p65** | #6956 | 1:250 | CST |
| **β-actin** | sc-47778 | 1:1000 | Santa cruz |

TLR: Toll-like receptor; MyD88: Myeloid differentiation factor 88; NF-κB: nuclear factor-kappa B.

**Supplemental Table 3**: Antibodies used for immunofluorescence.

| **Antibodies** | **Catalog No.** | **Dilution** | **Company** |
| --- | --- | --- | --- |
| **TLR4** | ab22048 | 1:100 | Abcam |
| **NeuN** | ab177487 | 1:1000 | Abcam |
| **GFAP** | ZA-0117 | 1:1000 | GSGB-BIO |
| **Iba1** | 019-19741 | 1:1000 | Wako |
| **Alexa Fluor555** | A31572 | 1:400 | LIFE |
| **Alexa Fluor488** | A21202 | 1:400 | LIFE |

Iba1: ionized calcium-binding adaptor molecule; GFAP: glial fibrillary acidic protein; NeuN: neuron nuclei.

**Supplemental Table 4**: Primer sequence for real-time PCR.

| **Gene** | **forward primer** | **reverse primer** |
| --- | --- | --- |
| **miR-93** | 5´-CAAAGTGCTGTTCGTGCAGGTAG-3’ |  |
| **TLR2** | 5´-TGCTTTCCTGCTGAAGATTT-3’ | 5´-TGTACCGCAACAGCTTCAGG-3’ |
| **TLR4**  **GAPDH** | 5´-CCGCTCTGGCATCATCTTCA-3’  5′-TCCATGACAACTTTGGCATC-3 ′ | 5´-TGGGTTTTAGGCGCAGAGTT -3’  5′-CATGTCAGATCCACCACGGA-3 ′ |
